# Combined Candida dubliniensis and Candida albicans Keratitis following a Chemical Injury

**DOI:** 10.1155/2019/7628126

**Published:** 2019-05-13

**Authors:** Justin Torosian, Thomas Mauger

**Affiliations:** West Virginia University Eye Institute, 1 Medical Center Drive, Morgantown, WV 26506, USA

## Abstract

*Candida dubliniensis* is an uncommon source of ocular infections and has only been reported in a single previous case of keratitis. This report documents the course of a combined* Candida dubliniensis* and* Candida albicans* keratitis following a chemical injury. Antifungal sensitivities of the two different* Candida* species are also demonstrated.

## 1. Introduction

Fungal keratitis is a challenging clinical condition. The diagnosis and initiation of appropriate treatment may be delayed due to the insidious nature of the disease. Once identification of the organism is obtained and specific treatment is initiated, the course of therapy is often prolonged and clinical outcome may be poor. Fungal keratitis may be caused by either filamentous or yeast species. Filamentous fungi are more commonly associated with refractive contact lens wear and ocular trauma while yeasts are more commonly encountered in cases of chronic ocular surface disease [1, 2].

## 2. Case Presentation

**Figure 1:**
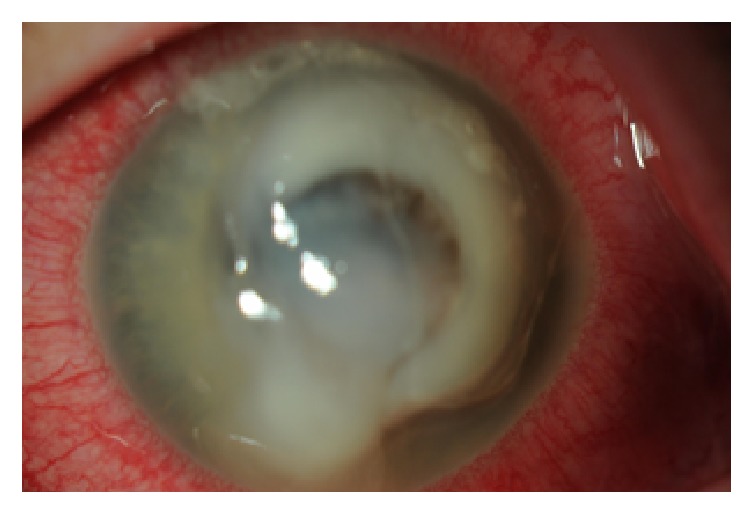
Central corneal epithelial defect with stromal infiltrate and hypopyon.

A 43-year-old female presented to the emergency department with a history of a liquid chemical exposure to the right eye with a household liquid cleaner containing 6% sodium hypochlorite. Examination demonstrated a central 5 mm corneal epithelial defect and diffuse conjunctival injection. The corneal stroma was edematous. The eye was treated with topical atropine 1% BID, prednisolone acetate 1% QID, tobramycin-dexamethasone ointment QHS, ofloxacin QID, doxycycline 100 mg by mouth BID, vitamin C 1 tab by mouth daily, and preservative-free artificial tears QID. The patient was seen two days later and a 3 mm central stromal infiltrate was noted. Fortified vancomycin 50 mg/ml and tobramycin 15 mg/ml were added topically every hour while awake. The patient was referred to our clinic two days later and found to have a 3 mm anterior stromal infiltrate with fluffy borders with an overlying 5 mm epithelial defect consistent with infectious keratitis. A one millimeter hypopyon was present (Figure 1). The cornea was cultured for bacteria and fungus. All steroid containing medications were stopped. Cultures were negative. The cornea remained unchanged over the next week. The patient was taken to surgery for a corneal biopsy, repeat corneal cultures, cryotherapy, and a conjunctival flap. These cultures grew* Candida dubliniensis* and* Candida albicans*. Sensitivities to antifungal agents were obtained (Table 1).

**Figure 2:**
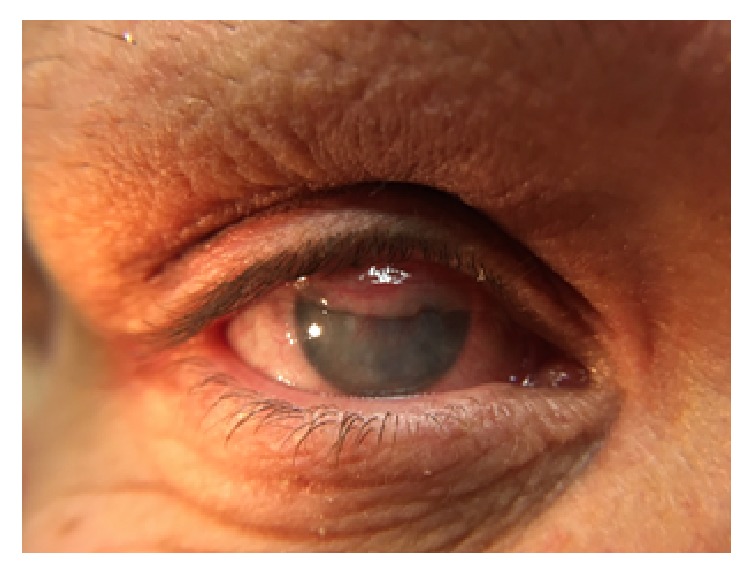
Corneal ulcer after Cryo treatment and conjunctival flap.

The eye was treated with hourly topical natamycin 5% and the infection resolved over the next three weeks (Figure 2). She is awaiting a corneal transplant due to resultant corneal opacity.

## 3. Discussion

To our knowledge,* C. dubliniensis *has been described only once previously in the literature as a cause of fungal keratitis and never in combination with* C. albicans*. In this previous case, the patient's condition worsened despite topical antifungal therapy and required cryotherapy, penetrating keratoplasty, and instillation of intracameral antimicrobial and antifungal agents, as well as partial conjunctival flap placement [3].

*C. dubliniensis *was first designated as a unique organism in the United Kingdom in 1995, where it was identified as a causative agent of oral candidosis [4]. More recently, it has been identified as an uncommon cause of fungemia in cancer patients undergoing treatment, though it remains far rarer than various other candida species [5].

Regarding ocular involvement,* C. dubliniensis* has been known to be a potential causative organism of endophthalmitis, described multiple times in the ophthalmology literature in North America since 2012 [6–8]. In this case presentation, multiple aspects of the clinical presentation as well as the innate properties of* C. dubliniensis* become of interest.* C. dubliniensis* is a novel cause of fungal keratitis that can be difficult to identify and treat but is felt to be less virulent than* C. albicans* and generally susceptible to available antifungal therapies.

*C. dubliniensis* has been shown to produce germ tubes and chlamydospores, which has typically been thought of as a property of* C. albicans*. However, this novel species has been shown to be less robust on warmer temperature cultures [4]. It is interesting that both* C. albicans* and* C. dubliniensis* were shown to have grown in this patient. Furthermore, the particular culture of* C. dubliniensis* isolated was found to be less susceptible to caspofungin, but more susceptible to 5 flucytosine, amphotericin B, fluconazole, and itraconazole, and equally susceptible to voriconazole compared to the* C. albicans *isolate.

In vitro,* C. dubliniensis *has been shown to develop resistance to fluconazole in a multifactorial fashion similar to* C. albicans*, including both upregulation of efflux transporters and mutations in the gene encoding for lanosterol demethylase [9]. Furthermore,* C. dubliniensis* has been shown to be less virulent than* C. albicans*, which has been postulated maybe in part due to lower efficiency in hyphae formation [10]. Interestingly, recent literature has illuminated that* C. dubliniensis* hyphae formation is limited in nutrient-rich media due to changes in expression of transcriptional regulatory protein UME6 [11].

Of interest, as opposed to the first reported case of* C. dubliniensis* keratitis, our patient had both* C. dubliniensis *and* C. albicans* isolated in the corneal biopsy culture. The previously reported case in the literature was referred to the authors for nonhealing corneal ulcer, but the initial mechanism of injury is unclear. In this case, the initial injury to the eye was a severe chemical burn from an alkaline solution, sodium hypochlorite. It is reasonable to consider that the severe disruption of corneal architecture allowed for the growth of multiple infectious organisms. Furthermore, it begs the question of how many cases of fungal keratitis first begin as multiorganism infections. It is unclear how commonly multiple candidal species coexist in other modes of infection; however,* C. dubliniensis *and* C. albicans* have been shown to do so in oral samples taken from children with dental caries [12]. Previously, murine models have shown an inability for* C. dubliniensis *to cause keratitis as a single organism in immunocompetent mice. However, there was an ability to cause keratitis in immunocompromised models [13]. Perhaps the severe chemical injury in our patient provided a relative lack of blood flow and therefore created isolation from the body's typical immune response.

Of note, there are several limitations to this case study. First is that the organisms were detected and isolated by corneal culture. Perhaps in the future, additional collateral information including histology or sampling of other sites, including the mouth, nares, and any eye medications the patient may have used, would be of interest. Finally, given the ubiquitous nature of various candida species, the possibility of a culture contaminant must be considered.

Fortunately, the previous reported case of* C. dubliniensis *keratitis had a positive outcome following instillation of intracameral antifungals and antibiotics as well as penetrating keratoplasty. Indeed, although* C. dubliniensis* keratitis is an uncommon cause of fungal keratitis, it tends to be less virulent of a species and remains largely sensitive to common antifungal agents.

## Figures and Tables

**Table 1:** Anti-Fungal Susceptibilities.

Anti-Fungal	Candida albicans	Candida dubliniensis
	MIC SUSCEPTIBILITY	MIC SUSCEPTIBILITY
	(mcg/mL)	(mcg/mL)
5 Flucytosine	0.5	0.06
Amphotericin B	0.5	0.225
Caspofungin	0.06	0.12
Fluconazole	0.25	0.12
Itraconazole	0.12	0.03
Voriconizole	0.008	0.008
